# Supplementary figures and images for: Chemistry, taxonomy and ecology of the potentially chimpanzee-dispersed Vepris teva sp.nov. (Rutaceae) endangered in coastal thicket in the Congo Republic
Source: PeerJ. 2022 Aug 23;10:e13926. doi: 10.7717/peerj.13926 (PMC9415428; doi:10.7717/peerj.13926)

**1H NMR spectra for compounds 1 - 6**


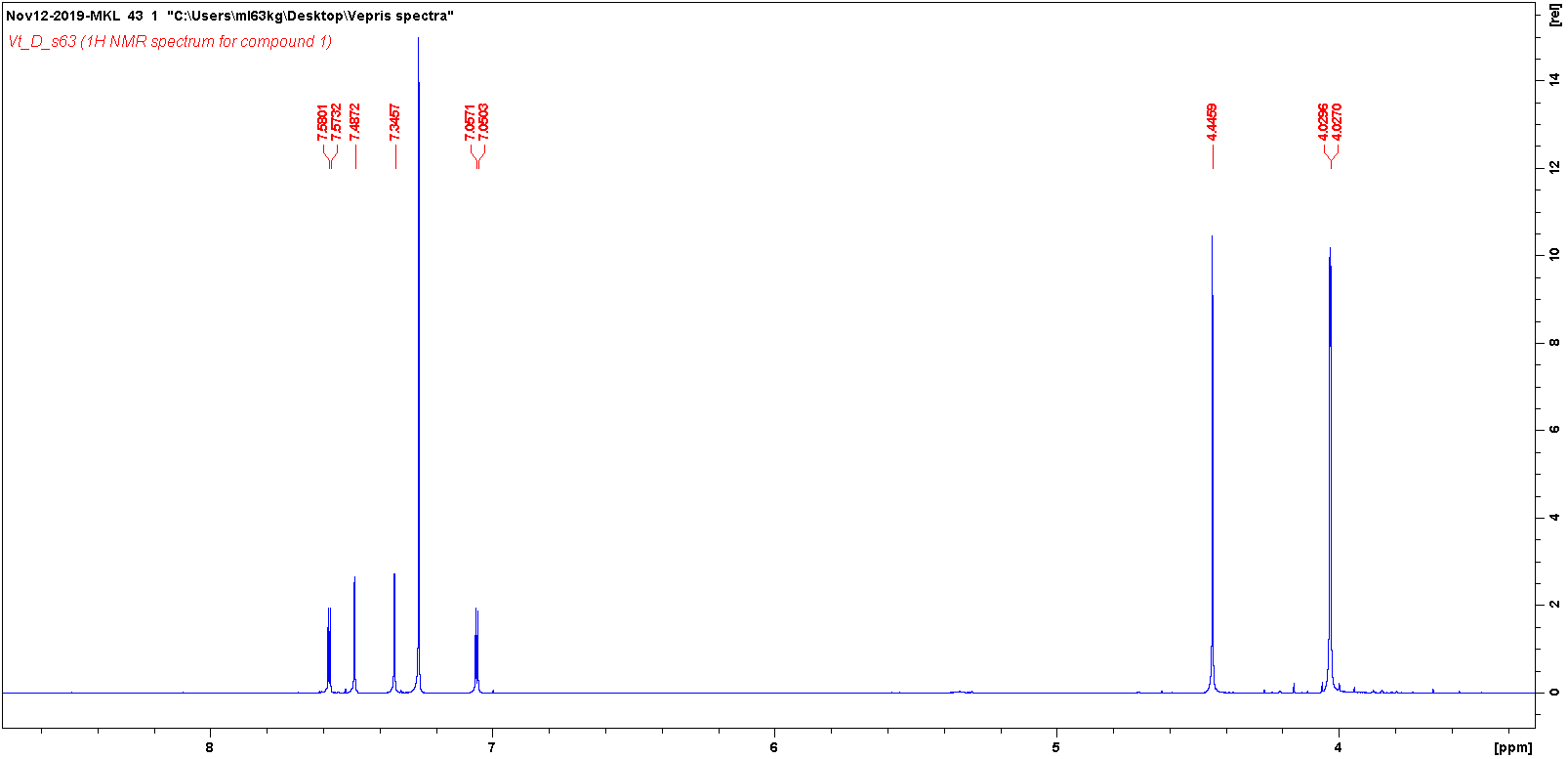


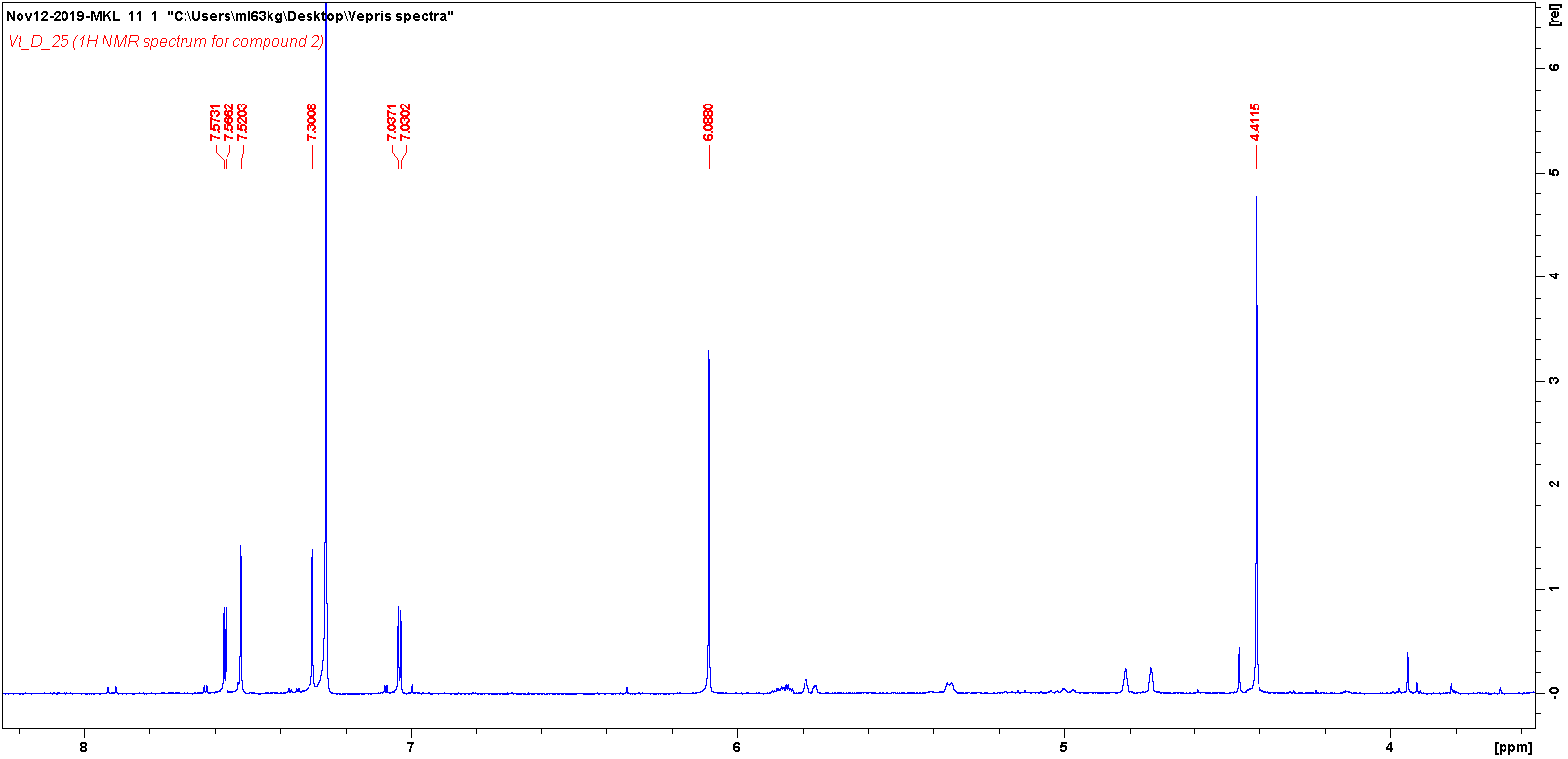


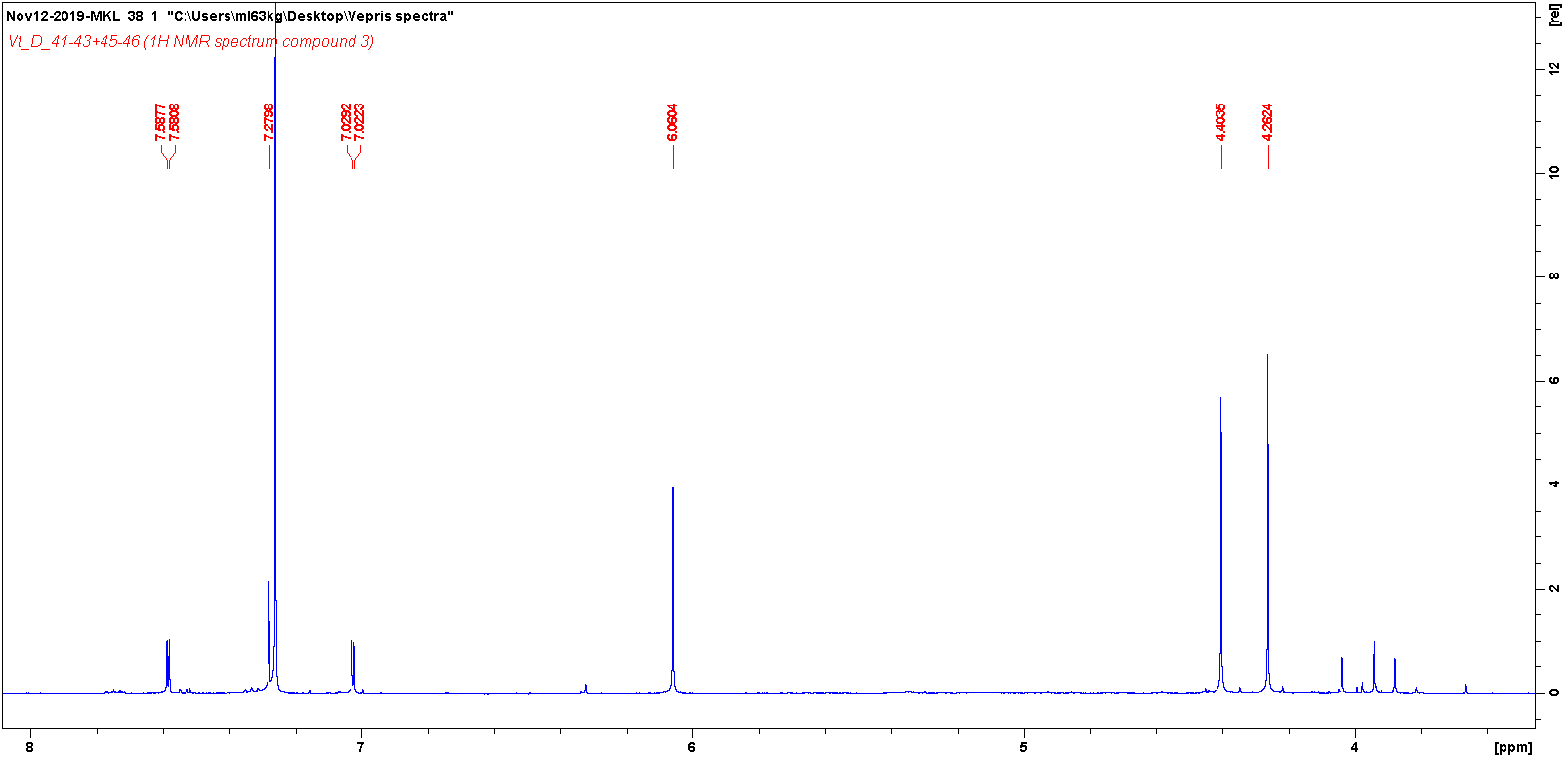


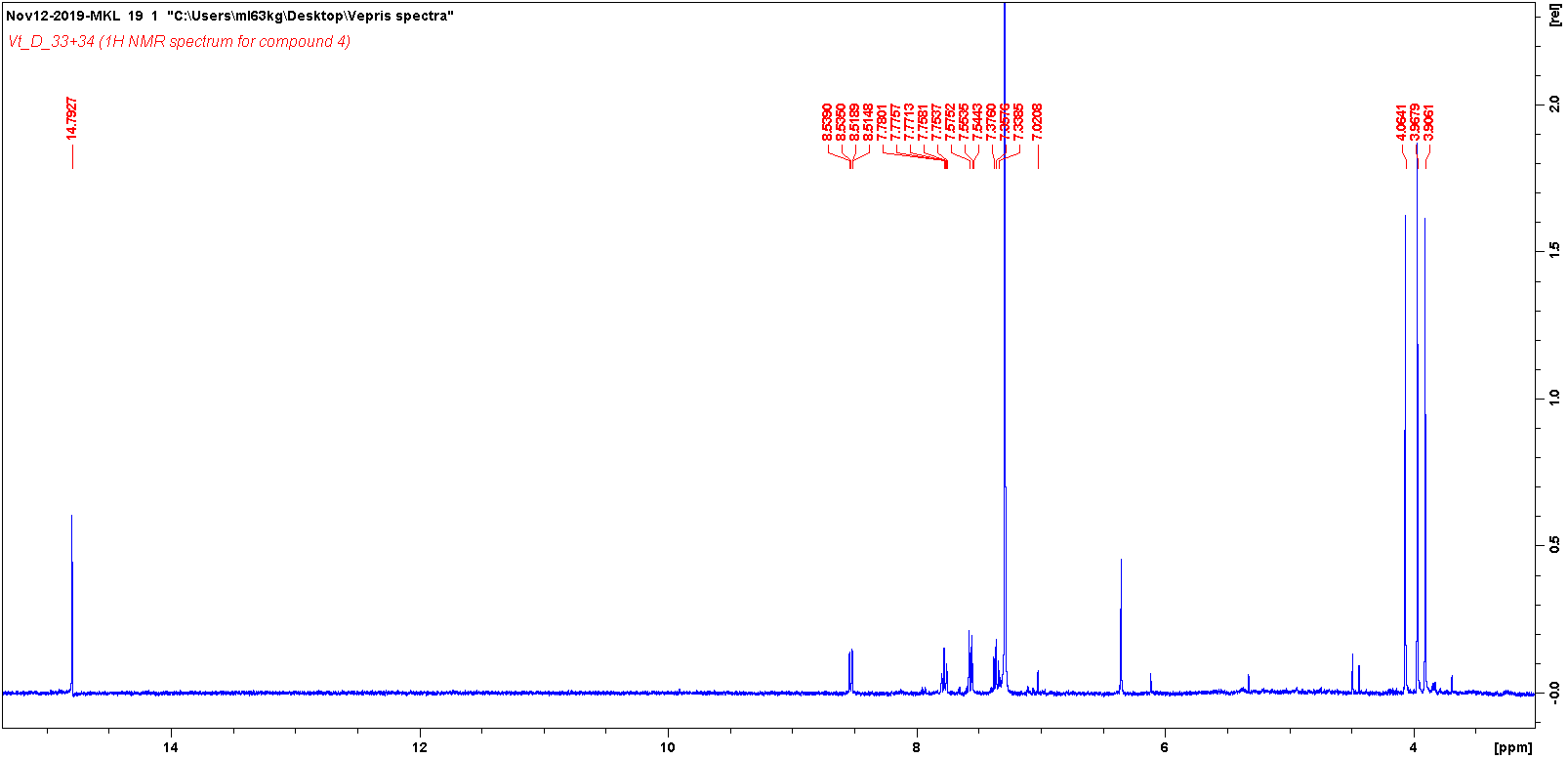


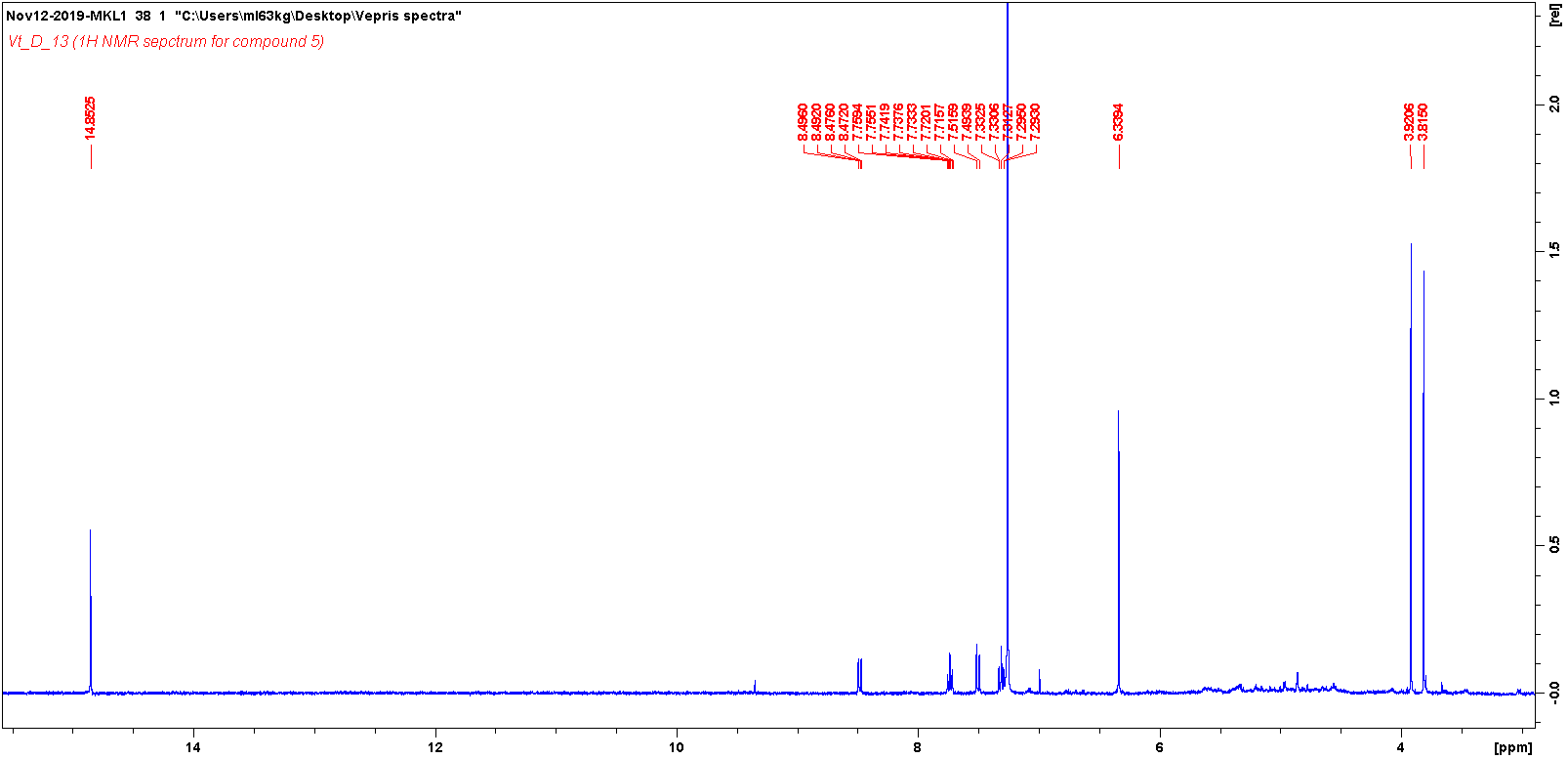


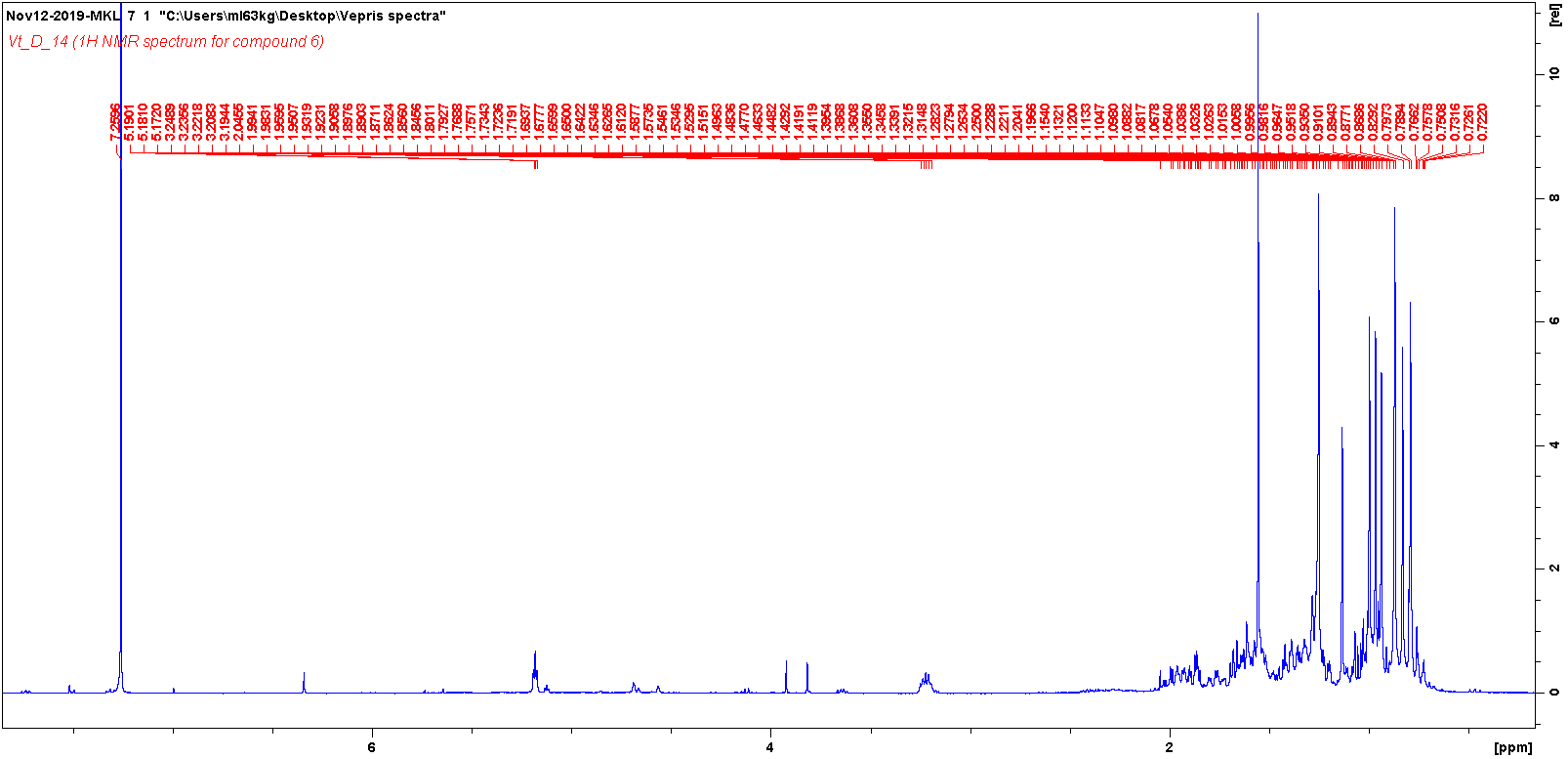

Supplement: Supplemental Information 1 [file peerj-10-13926-s001.docx]
